# Effects of Maternal Exposure to Lamotrigine during Pregnancy on Gonadal Toxicity in Fetus Rats

**DOI:** 10.31661/gmj.v9i0.1557

**Published:** 2020-10-03

**Authors:** Hoda Aryan, Shabnam Movassaghi, Amir Ghasemi, Roksana Darabi

**Affiliations:** ^1^Department of Internal Medicine, Semnan University of Medical Sciences, Semnan, Iran; ^2^Anatomy Department, Islamic Azad University, Tehran Medical Sciences, Tehran, Iran; ^3^Medical Students’ Scientific Association (MSSA), Tehran Medical Sciences, Islamic Azad University Tehran, Iran; ^4^Young Researchers and Elite Club, Tehran Medical Sciences, Islamic Azad University, Tehran, Iran; ^5^Department of Obstetrics and Gynecology, Islamic Azad University, Tehran Medical Sciences, Tehran, Iran

**Keywords:** Lamotrigine, Pregnancy, Embryo, Testicular, Ovary

## Abstract

**Background:**

Lamotrigine is one of the newest antiepileptic drugs that is used as one of the most common treatments in pregnancy. Since the investigation of the teratogenic effects of lamotrigine is very limited and there is no report of its teratogenic effects on fetal gonads, we aimed to investigate the teratogenic effects of lamotrigine on embryonic gonads.

**Materials and Methods:**

This study was performed on nine female Wistar female rats (8 weeks, weighing 180-200 mg). At first, the animals were inspected regularly by the preparation of vaginal smear and in the estrus phase in separate cages of mating, and after observing the vaginal plaque, were randomly divided into three groups (n=3). Control group did not receive any treatment. In the lamotrigine group (20mg/kg), and the vehicle group (same volume of normal saline) were injected intraperitoneally from days 8 to 13 of pregnancy. On day 20, animals were anesthetized by sodium pentobarbital (40 mg/kg), and embryos were extracted through laparotomy. First, fetuses were weighed, and their height (crown-rump length) was measured. Then the gonads of the fetuses were removed and, stained with H & E, and examined by optical microscope.

**Results:**

Our results showed that in the lamotrigine group, the number of seminiferous tubules and Sertoli cells in the male embryos and the number of oocytes in the female embryos decreased significantly compared to the control and vehicle groups (P≤0.05).

**Conclusion:**

The results of this study showed that treatment with 20 mg/kg lamotrigine in mothers during pregnancy could cause damage to fetal gonads.

## Introduction

Epilepsy as an important neurological disorder could affect 5 per 1000 people. It is temporary and reversible impairment in the function of neurons. It includes some syndromes, which the key aspect of all of them is the tendency to seizure without stimulating actions [1]. According to statistical studies, about 1.1 million women suffer from epilepsy, and the prevalence of epilepsy in pregnancy is 3.3 per 1,000 pregnancies, and most of these pregnancies end up without any complications [2]. Different antiepileptic drugs are used to treat pregnant mothers suffering from epilepsy. These drugs are classified into two groups in terms of the risk of their use during pregnancy. The first group is the older drugs, and extensive studies have been conducted on them. The second group is a newer type of drugs in which there is limited information on them [2]. Previous studies demonstrated some common anti-epileptics (e.g., sodium valproate, barbiturate, carbamazepine, etc.) could lead to potential anomalies including neural tube defects, hypoplasia and/or aplasia of phalanges and nails, cleft palate and lip, cardiac abnormalities, cranial-facial defects( such as microcephaly, epicanthic folds, upward palpebral fissure, mandibular hypoplasia, short nose, long philtrum, ocular anomalies, reduced growth, and developmental delay [3-5]. Lamotrigine is one of the newest anti-epileptic drugs that its using started in 1992 by very few patients [6]. It is one of the most effective medications used for the treatment of epilepsy and some psychiatric disorders like bi-polar disease [6]. Lamotrigine is categorized as phenyl thiazine drugs with the molecular formula of C6H7N5. This drug is easily absorbed through the digestive system. However, research has shown that this drug passes easily through the placenta and is secreted during lactation [6]. Conducting studies on anomalies caused by lamotrigine is very limited. Despite some clinical studies; little information is available on its safe dose and type of anomalies [5,7]. In a recent study, the incidence of its abnormality was reported by 2.9%, but no specific pattern was observed [7]. Also, animal research is limited, and in most studies in which has been used orally, no report of teratogenic cases has not been providing [7]. Since studies on teratogenic effects of lamotrigine are very limited and there is no report available on its effects on the fetal gonads, we aimed to investigate the teratogenic effects of lamotrigine on fetal gonads of rats.

## Materials and Methods

 This experimental study was carried out at the Medical Sciences Research Center of Islamic Azad University, Tehran Medical Branch. First, nine Wistar female rats (obtained from Pasteur Research Institute, Iran) with an age of 8 weeks before starting the study were kept at 23-25 ° C, and 12 hours of the light-darkness cycle for one week to adapt to the laboratory conditions. The female animals were regularly examined by the preparation of vaginal smear, and mating was done in separate cages in the estrus phase. After observing the vaginal plaque, the rats were randomly divided into three groups (n=3 pregnant rats) at gestational days (GD) 1–20 as follows:

 1. Control group: No treatment was provided for the animal of this group.

 2. Lamotrigine group: 20 mg/kg of lamotrigine (Sigma, Germany) was injected intraperitoneally (i.p) for six days (from the GD8 to GD13)

 3. Vehicle group: the same volume of normal saline was injected i.p for six days (from the GD8 to GD13

**Figure 1:**
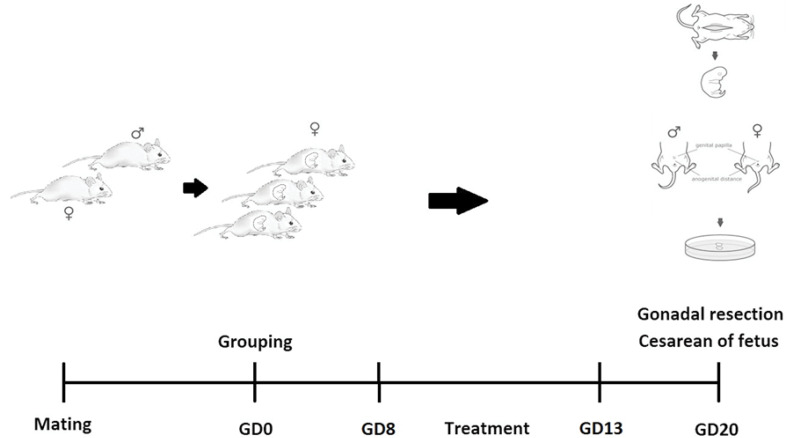
Timeline of study

 Then, on the 20th day of pregnancy, the rats were removed by cesarean section, and their fetuses were removed (Figure-1). Immediately after removing the fetuses, the number of fetuses in each group was counted. Then, they were weighed using a special scale, and the crown-rump length (CRL) was recorded. Moreover, the apparent anomalies of the fetus and their placenta were examined. The gonads (ovarian and testis) were dissected from the fetuses; then were processed for hematoxylin and eosin staining. In brief, ovaries and testis were fixed in cold 4% paraformaldehyde for 24 hours, embedded in paraffin, and serially sectioned to a thickness of 5μm. The sections were examined by an optical microscope (Olympus Optical, Tokyo, Japan) with 400 × magnification. Eight photomicrographs were prepared from each sample, which three photomicrographs with the minimum distance of 40 microns were randomly selected. In testis specimens, the number of seminiferous tubules in each field was counted, and their mean was reported. To count the Sertoli cells, five seminiferous tubules were randomly selected in each field, and the mean number of Sertoli cells was reported. In the ovaries samples, the number of oocytes was counted by Imaging-Pro-Plus 6 software (Media Cybernetics Inc., USA). All results are expressed as mean ± standard deviation (SD). Data were analyzed using an analysis of variance (ANOVA) and Tukey with SPSS v.20 software (IBM, New York, USA). Values of P<0.05 were considered statistically significant.

## Results

###  1. Examining the Appearance of Fetuses and Their Placenta

####  1.1. Number of Fetuses and Skeletal-Placental Anomalies

 Out of 33 fetuses in the control group, 33 fetuses in the vehicle group, and 43 fetuses in the lamotrigine group, male to female ratio was 19/14, 16/17, and 22/21, respectively. Also, fetuses have normal morphology, and their placentas had no anomaly.

####  1.2. Weight of Fetuses

 The mean weight of female fetuses in the lamotrigine group (3.34 ± 0.2 g) was lower than that of the control group (3.85 ± 0.18 g, Table-1). Data analysis showed that this difference in weight of female fetuses between the lamotrigine group and vehicle group and the control group was significant (Table-1, P≤0.001). Moreover, the mean weight of male fetuses in the lamotrigine group (3.17±0.18 g) was lower than that of the control group (4.1 ± 0.61 g). Data analysis showed that this difference between the lamotrigine group and the vehicle and the control groups was significant (Table-2, P≤0.001).

####  1.3. CRL of Fetuses

 The mean CRL of female fetuses in the lamotrigine group was 2.6 ±0.09 cm (Table-1), while this value was higher than in the control and vehicle groups, which this difference was statistically significant (Table-1, P≤0.001). Similarly, the mean CRL of male fetuses in the lamotrigine group was lower than those in control and vehicle groups, in which this difference was statistically significant (Table-2, P≤0.001).

###  2. Morphology of Seminiferous Tubules and Sertoli Cells

**Figure 2:**
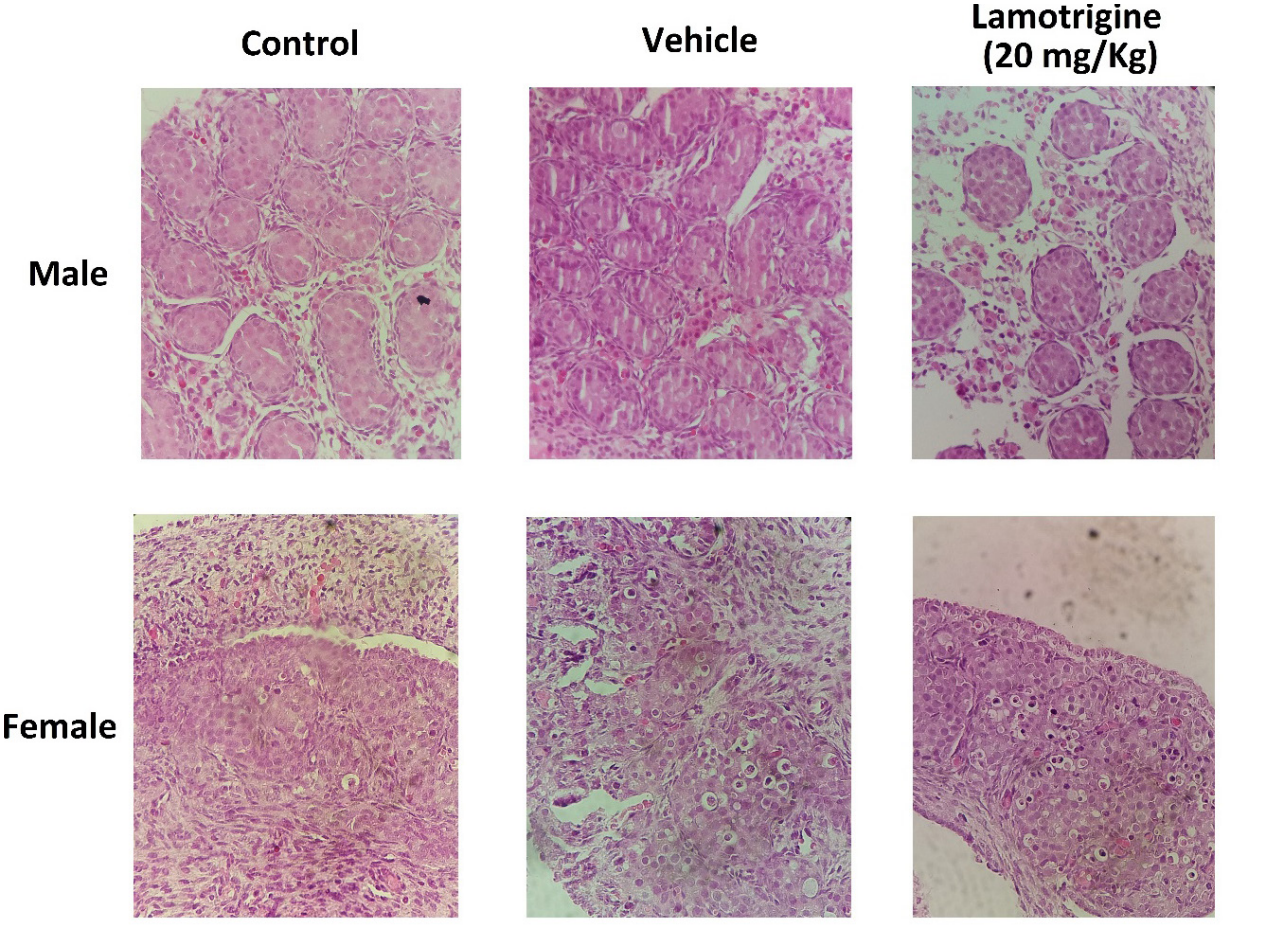
Photomicrograph of fetuses’ gonads. Normal seminiferous tubules with Sertoli cells was observed in control and vehicle groups. Treatment with lamotrigine significantly decreases the number of both seminiferous tubules and Sertoli cells. Also, in the female fetuses, the number of primordial oocytes was in control and vehicle groups was more than lamotrigine group. (Hematoxylin and eosin staining, original magnification ×400)

**Figure 3:**
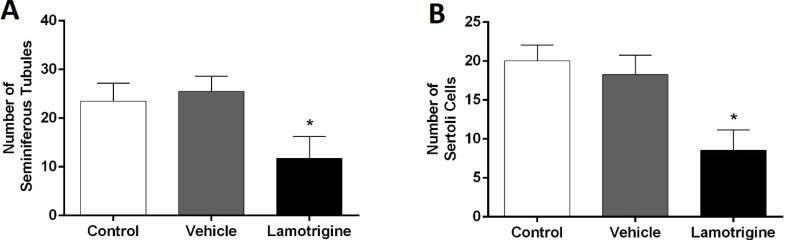
The number of seminiferous tubules ( **A**) and Sertoli cells ( **B**) in the lamotrigine group was significantly lower than the control and vehicle groups. *P≤0.001 vs. control group

 In the histological examination of testicular tissue in the control group, seminiferous tubules with normal appearance along with Sertoli cells were observed (Figure-2). Histological characteristics of the vehicle group were similar to those of the control group, and there was no significant difference between the two groups. However, in the group that had exposure with lamotrigine during the fetal period, degeneration of the seminiferous tubules, reduced number, and degeneration of the Sertoli cells were observed, which these changes in the number and morphology were significant in comparison to the control group (Figure-2 and Figure-3).

###  3. Morphology of Oocytes

**Figure 4:**
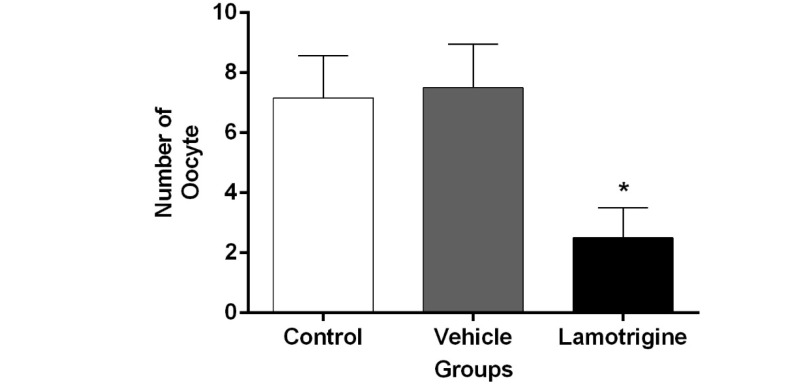
The number of the primordial oocyte in the lamotrigine group was significantly lower than the control and vehicle groups. *P≤0.001 vs. control group

 In the histological examination of ovarian in the control group, oocytes were observed with normal appearance (Figure-2). The histological characteristics in the vehicle group were similar to those of the control group, and there was no significant difference between them in terms of morphology and cell count (Figure-4, P≤0.001). However, the number of oocytes in the lamotrigine group significantly decreased compared to that in the control group.

## Discussion

Nowadays, the use of anti-epileptic drugs such as lamotrigine during pregnancy is a major constraint and a major challenge in treating the patients with epilepsy due to possible adverse effects on the fetus. Many studies have shown that the use of these drugs during pregnancy has been associated with skeletal, developmental, neural, cardiac and even fetal deaths [8-10]. However, there is limited information on the probable side effects of lamotrigine on fetus’ gonads during pregnancy. In other words, the present study was the first study, which evaluates the teratogenicity effects of lamotrigine on fetal gonadal tissues.

###  Effect of Lamotrigine on Weight, CRL, and Apparent Anomalies of the Fetuses

Our results showed that receiving 20 mg/kg of lamotrigine in the prenatal period did not cause skeletal and fetal anomalies. However, the CRL and weight of male and female fetuses in the lamotrigine group were significantly lower than those of the control and vehicle groups, indicating reduced intrauterine growth or small for gestational age (SGA). In a study conducted by Prakash *et al*. in 2016 on the maternal and fetal effects of lamotrigine, the results showed an incidence of SGA in two newborn infants. However, fetal anomalies were not identified [10]. In another study conducted by Tennis *et al*. in 2002, infants of mothers who were treated with lamotrigine in the first trimester of pregnancy were born with a higher risk of fetal anomalies [11]. In other words, in this group of infants, although anomalies resulting in stillbirth were not seen, skeletal defects were reported in some of them. However, this rate was lower compared to other drugs, such as sodium valproate. The study conducted by Rahmani *et al*. (2005) on mice showed that lamotrigine was probably a risk factor for the incidence of skeletal abnormalities in the fetus of mice receiving lamotrigine during pregnancy [7]. The results of this study showed that the height of fetuses whose mothers received lamotrigine during pregnancy was significantly lower than that of the control and carrier groups. In the survey conducted by Sathiya *et al*. in 2014 on the effects of lamotrigine on rats, the mean length of fetuses whose mothers were treated with lamotrigine significantly decreased [12]. Therefore, according to previous studies, the risk of low birth weight and skeletal abnormalities in fetuses whose mothers used lamotrigine during pregnancy is higher than that of the normal population, which these results are consistent with those of the current study.

###  Effect of Lamotrigine on Ovaries of Fetuses

Results of our study indicated that the number of oocytes of fetuses in the lamotrigine group was significantly lower than that of the control and vehicle groups, indicating significant damage to the gonadal tissue of the fetuses. As stated before, no study was conducted on the investigation of the effects of lamotrigine on gonads of the fetuses. However, in a study conducted by Røste *et al*. (2003) on the long-term treatment effects (90 days) of 5 mg lamotrigine on adult female rats, the results showed that the number of oocytes and secondary follicles in the group receiving drug decreased significantly compared to that of the control group [13]. Hence, as with adult rats, it seems that lamotrigine causes early ovarian failure and reduced fertility in the fetuses whose mothers received drug by reducing oocytes and follicles of the ovaries.

###  Effect of Lamotrigine on the Testis of Fetuses

The results of the current research showed that the number of seminiferous tubules in the fetuses of the lamotrigine group decreased significantly compared to that of control and vehicle groups. In a study conducted by Khezri Motlagh *et al*. (2016) on the effects of lamotrigine on testicular tissue and male hormones levels in adult rats, the results showed a significant reduction in testicular volume and weight as well as the level of testosterone and FSH in the group treated with 400 mg of lamotrigine, compared to the control group [14]. In another study conducted by Røste *et al*. in 2003 on the effects of long-term effects of lamotrigine on testicular tissue in adult rats, the results showed that the number of seminiferous tubules in the drug-receiving group decreased significantly in comparison to that of the control group [13]. This result is also in line with that of the current study. In a study conducted by Daoud *et al*. (2003) on the effects of lamotrigine and vigabatrin on testicular tissue and fertility rate in adult rats, the results showed a significant reduction in the number of seminiferous tubules in the lamotrigine group compared with the control group. Also, the number of Leydig and Sertoli cells also decreased significantly in the lamotrigine- treated group [15]. In the current study, the number of Sertoli soles in the fetuses of the lamotrigine group decreased significantly compared to that of the control and carrier groups, which is consistent with the results of previous studies. Although previous studies focused on the effects of lamotrigine on the reproductive system in adult mice, our results suggest that its destructive effects on fetuses which had contact with the drug during pregnancy were similar and even more. In a study conducted by Ohman *et al*. in 2000 on nine pregnant women and ten infants, the results of the study showed that at the time of delivery, the plasma concentration of lamotrigine was similar to that in the umbilical cord and lactation. It means that the drug passes through the placenta and it is found in the lactation [16]. It seems that lamotrigine can pass through the placenta during the fetal period and applies its toxicity on the gonads and finally affect the rate of fertility in the future.

## Conclusion

 The results of this study showed that treatment with 20 mg lamotrigine in mothers during pregnancy could damage fetal gonads. In other words, lamotrigine seems to reduce fertility capacity by direct damage to seminiferous tubules in male fetuses and by reducing the number of oocytes in female fetuses. However, it is suggested that further studies in animal models as well as clinical studies to be conducted to investigate the possible toxic effects of lamotrigine on gonads.

## Conflict of Interest

 There is no any conflict of interests.

**Table 1:** Effects of Lamotrigine of CRL and Weight of Male Fetuses

**Variables**	**Control**	**Vehicle**	**Lamotrigine**	**P-value**
**Weight (g)**	3.85±0.18	3.7±0.19	3.63±0.28	0.001
**CRL (cm)**	3.06±0.11	3.06±0.09	2.6±0.09	0.001

**Table 2:** Effects of Lamotrigine of CRL and Weight of Female Fetuses

**Variables**	**Control**	**Vehicle**	**Lamotrigine**	**P-value**
**Weight (g)**	4.16±0.61	3.82±0.28	3.72±0.57	0.001
**CRL (cm**	3.15±0.11	3.05±0.09	2.96±0.22	0.001
